# More extensive lymphadenectomy in colon cancer; how far are we willing to go for a biomarker?

**DOI:** 10.1007/s10151-020-02239-0

**Published:** 2020-05-25

**Authors:** N. P. M. Brouwer, N. Hugen, I. D. Nagtegaal

**Affiliations:** 1 Department of Pathology, Radboud University Medical Centre, Geert Grooteplein Zuid 10, 6525 GA Nijmegen, The Netherlands; 2 Department of Surgery, Radboud University Medical Centre, Nijmegen, The Netherlands

## History

Metastatic disease is the main cause of death in colorectal cancer (CRC) patients. Staging of CRC is essential to predict prognosis, provide optimal treatment, and ultimately decrease mortality. Lymph-node metastases (LNM) are an unfavorable hallmark in CRC, since they are generally considered to form the gateway to distant metastases. Lymph-node metastases have the highest impact on treatment decisions. Suspicion of LNM on imaging might indicate the need for neoadjuvant therapy in rectal cancer, and when LNM are found in the resected specimen, this is an indication for adjuvant therapy in colon cancer patients. The focus on LNM as a hallmark in cancer spread has led to extensive efforts to improve both clinical and pathological lymph-node staging and quality standards that include a minimum of assessed lymph nodes [1]. Furthermore, new surgical techniques have been developed to optimize lymphadenectomy, such as the technique of total mesorectal excision (TME) [2]. Hohenberger et al. proposed the counterpart of TME for colon cancer, and complete mesocolic excision (CME) with central vascular ligation (CVL), in which the aim, especially with CVL, is to improve survival by increasing the lymph-node yield [3].

However, the emphasis on LNM for cancer spread has been challenged in recent years with the discovery of other forms of locoregional spread that may be stronger prognostic factors (e.g., vascular invasion, perineural growth, and tumor deposits) [4, 5]. Moreover, clonal analyses have enabled further determination of the origin of metastases and their relation with LNM [6]. Although surgery forms the cornerstone in curative CRC treatment, it is crucial to determine what we aim to achieve by more extensive surgical techniques, and how far we are willing to go in harvesting more lymph nodes at the cost of increased morbidity.

## Lymph nodes: a gateway to distant metastases or a biomarker?

Lymphadenectomy is thought to serve multiple purposes. First, when following the paradigm of sequential progression, in which LNM would be the gateway to distant metastases, removal of the LNM may be therapeutic and may improve the survival of patients. Indeed, removal of lymph-node tissue according standardized surgical techniques like TME have led to improved survival rates [7]. It is, however, unknown whether this is specifically caused by the removal of LNM or other structures that are in close proximity to lymph nodes. Furthermore, there is evidence, showing that LNM are not the gateway to distant metastases per se in a large proportion of CRC patients. Population-based studies show that 40–60% of metachronous metastasis develop in patients without LNM [8]. Also, Knijn et al. showed that the distribution of metastases in the liver or lung is equal for patients with and without LNM [9]. These studies support the hypothesis that metastatic spread can also occur independently of lymphatic spread, particularly to the liver and lung. In 2017, the study by Naxerova et al. led to a paradigm shift, by studying the evolutionary relationship between primary CRC tumors, LNM, and distant metastases. In 65% of the cases, there was no evidence of distant metastases arising from LNM [6]. Despite the fact that this was a study with a small number of patients and mostly liver metastases, it could mean that over two-thirds of distant metastases do not originate from LNM.

Second, since LNM are a strong predictive and prognostic factor, lymphadenectomy enables staging to optimize further treatment of patients. However, LNM are no longer the only or the strongest prognostic factor for metastatic disease in CRC. Several new prognostic factors have been discovered, including extramural vascular invasion (EMVI) and tumor deposits (TD). EMVI is associated with decreased survival and adds prognostic value to stratification by LNM in the prediction of liver and lung metastases. TD have significantly more prognostic power in predicting liver, lung, and peritoneal metastases compared with LNM. [10] It is conceivable that both EMVI and TD form the actual gateways to metastatic disease [10, 11].

Taking the current evidence into account, it can be questioned whether considering LNM as the only gateway to distant metastases in CRC is justified. It is likely that LNM are one of several gateways to distant metastatic spread, and that they function in large part as one of several biomarkers (i.e., as a sign of advanced disease).

## Extramural vascular invasion and tumor deposits: gateways to distant metastases

During the investigation of locoregional cancer spread, EMVI and TD have emerged as promising additional gateways. Venous invasion in CRC beyond the muscularis propria is called EMVI. EMVI creates access to an anatomic highway of the draining vasculature of the bowel, and thereby increases the risk of hematogenous metastatic spread. The increased risk of hepatic metastasis in the presence of EMVI supports the hypothesis that EMVI could form a hemodynamic pathway to distant metastatic disease [12]. Currently, vascular invasion is incorporated in guidelines as a histologic risk factor in colon cancer, for which adjuvant therapy could be considered.

TD are aggregates of tumor cells in the fat surrounding the bowel (i.e., the mesocolon and mesorectum) which are present in 20–25% of patients. They have been incorporated in TNM staging over the years, being considered lymph nodes (or not) and stratified according to size, contour or presence of other histological structures [10]. However, grouping TD together with LNM is incorrect based on their different prognostic value as well as distinct biology. TD can develop from multiple origins, including perineural, perivascular, intravascular, and lymphatic invasion, giving them access to multiple anatomic highways, and therefore possibly increasing their metastatic capacity.

Based on the prognostic evidence and their access to anatomic highways, it has been hypothesized that EMVI and TD could form additional gateways to LNM in causing distant metastases. The removal of EMVI and TD forms an alternative explanation for the survival advantage of patients undergoing TME or CME. The vast majority of TD are located in close proximity to lymph nodes, and are removed as a byproduct of the conventional lymphadenectomy [13]. However, it is important to note that there are currently no studies that report on the proximity of EMVI or TD to the additional lymph nodes removed with CVL as proposed by Hohenberger et al.

## The rationale behind extended lymphadenectomy: is it still up to date?

As insights regarding the mechanisms of cancer spread have developed over time, it is essential to evaluate whether the rationale behind promoting surgical resection beyond CME is valid.

The surgical technique proposed by Hohenberger et al. consists of two aspects: the dissection along the mesocolic plane (i.e., CME), and CVL. The rationale behind CME is understandable, since LNM as well as other potential gateways (e.g., EMVI and TD) lie within the mesentery. Resection of these gateways interrupts metastatic spread and can thereby improve survival. The idea that this type of resection represents higher surgical quality is widely accepted and it is nowadays standard of care for many CRC surgeons [14].

**Fig. 1:**
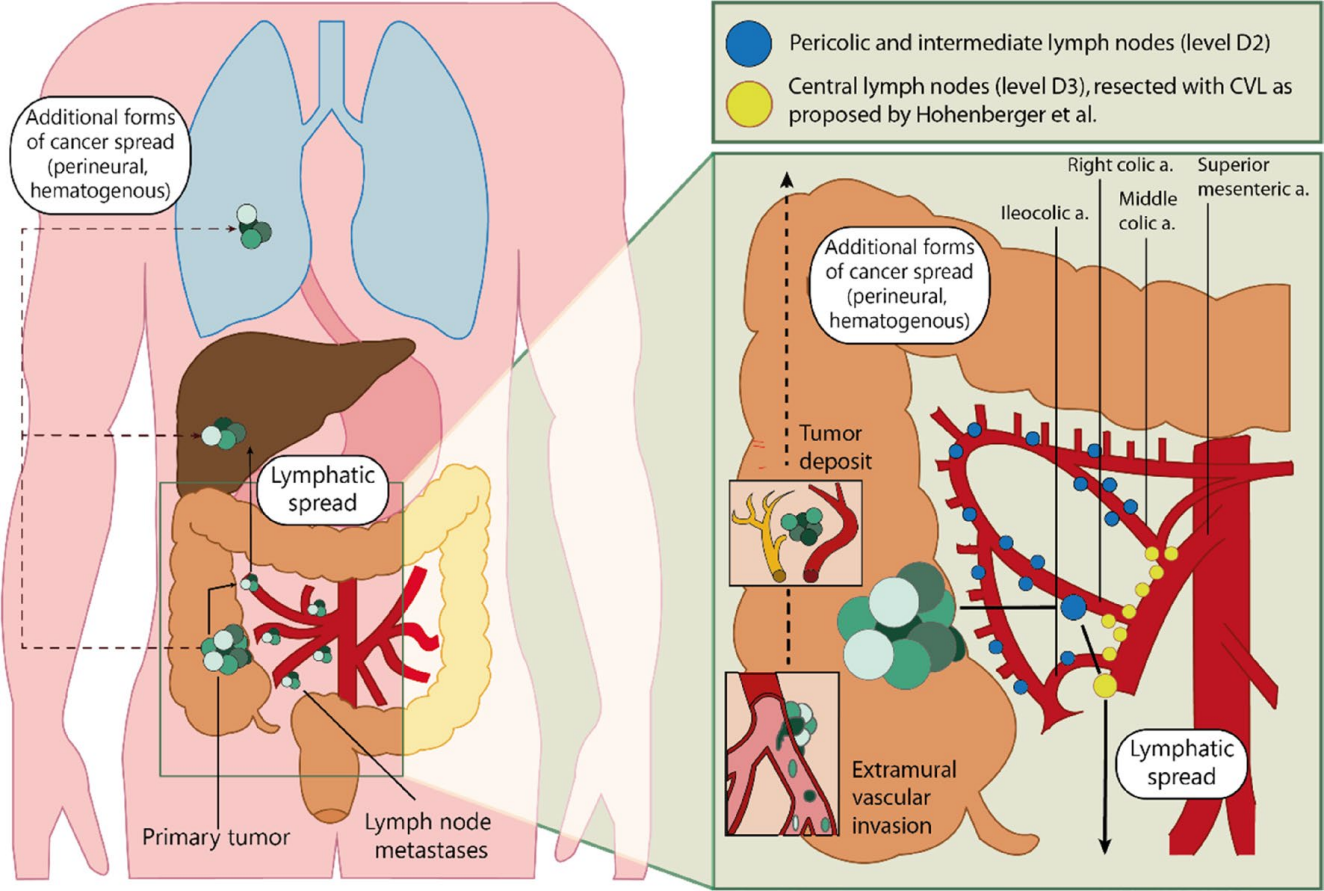
Different pathways of colon cancer spread and the effect of central vascular ligation (CVL). CVL is focused on resecting more lymphoid tissue in the form of central lymph nodes along the mesenteric arteries. However, as indicated by the dotted line, the pathways to distant metastases accessed by EMVI and TD are not increasingly interrupted by CVL compared with conventional surgery with D2 lymphadenectomy

In contrast, there are several issues regarding CVL that should be addressed. First, the definition of CVL is not undisputed. CVL refers to the ligation at the root of the supplying vessel to a given colonic segment. However, the subsequent extent of lymph-node dissection at the root is variable. Vascular ligation in combination with a lymphadenectomy at the root of the main feeding vessel is called a D2 resection. For a right-sided hemicolectomy, the supplying vessels are ligated right of the superior mesenteric vein and lymphoadipose tissue on the superior mesenteric vein is left intact. It has been demonstrated that vascular ligation at this level is associated with a high variability in residual arterial length [15]. A D3 dissection represents an extended lymphadenectomy that includes lymph nodes along the root vessel. This means that the vessels are ligated at the root of both the superior mesenteric vein and superior mesenteric artery and lymphoadipose tissue along the anterior aspect of the superior mesenteric vein/artery is dissected. The surgical procedure as proposed by Hohenberger et al. is considered more extensive than the D3 standard, mainly because of an increased longitudinal resection margins [16]. Second, the rationale behind CVL with a more extensive lymphadenectomy is less clear compared with CME. Promoting a more extensive resection by performing CVL is based on the belief that a more extensive lymphadenectomy has both additional therapeutic and prognostic benefits compared with CME alone. However, both of these arguments can be debated. When looking at the therapeutic effect of CVL, the evidence is conflicting. Some studies show improvement in recurrences as well as disease-free survival (DFS), while a systematic review showed that DFS did not improve after CME with CVL after exclusion of the results from Hohenberger’s study [3, 17, 18]. CME with CVL leads to higher postoperative mortality, as well as more intraoperative and postoperative complications [19]. Thus, the current evidence is insufficient to prefer a CVL over the conventional CME. Not only is the clinical evidence conflicting, the rationale behind the therapeutic effect of CVL can be questioned. CVL is focused on harvesting more lymph nodes, while evidence suggests that LNM are not the only way to distant metastases, with EMVI and TD as additional pathways. One can argue that EMVI or TD might also be resected to a larger extent when CVL is performed. However, EMVI as well as TD provide the opportunity for hematogenous spread, and once this has occurred, it cannot be stopped by resecting lymphoid tissue (Fig. 1). Since it is currently impossible to assess what pathway causes distant metastases in which patient, it is unclear what proportion of patients will benefit from resecting more lymphoid tissue with CVL in terms of prevention of metastatic spread and improved survival.

The presence of LNM has the most impact on prognosis in the current TNM staging system. However, extending the lymphadenectomy would only give extra prognostic information, and thus upstaging, if LNM would be found at the level of CVL and not closer to the primary tumor. This type of skip metastases seems to occur in only 2% of CRC patients, leading us to question the extra prognostic evidence that harvesting more distant lymph nodes would yield [20]. Furthermore, the gap in our knowledge about the presence of EMVI or TD at the level of CVL as well as their precise role in metastatic spread has to be filled, before the presence of these biomarkers could improve staging.

## Conclusions

There is little debate about the rationale behind CME and it is already widely used in daily practice. However, the implementation of CVL is still debated. The lack of a standardized definition of CME with CVL makes it difficult to interpret the results from studies reporting on the outcome of this technique. Furthermore, the role of LNM as the only hallmark in CRC metastasis is no longer undisputed with the finding of other possible gateways to distant metastases, such as EMVI and TD. With these insights, the additional value of a more extensive lymphadenectomy such as CVL proposed by Hohenberg et al. becomes a less attractive option for staging. Instead of focusing on LNM, future research as well as advances in treatment or staging should try to provide a better understanding of CRC spread that goes beyond the significance of LNM.

## References

[1] Resch A, Langner C (2013). Lymph node staging in colorectal cancer: old controversies and recent advances. World J Gastroenterol.

[2] Heald RJ (1988). The 'Holy Plane' of rectal surgery. J R Soc Med.

[3] Hohenberger W, Weber K, Matzel K, Papadopoulos T, Merkel S (2009). Standardized surgery for colonic cancer: complete mesocolic excision and central ligation—technical notes and outcome. Colorectal Dis..

[4] Knijn N, Mogk SC, Teerenstra S, Simmer F, Nagtegaal ID (2016). Perineural invasion is a strong prognostic factor in colorectal cancer: a systematic review. Am J Surg Pathol.

[5] Knijn N, van Exsel UEM, de Noo ME, Nagtegaal ID (2018). The value of intramural vascular invasion in colorectal cancer—a systematic review and meta-analysis. Histopathology.

[6] Naxerova K, Reiter JG, Brachtel E, Lennerz JK, van de Wetering M, Rowan A (2017). Origins of lymphatic and distant metastases in human colorectal cancer. Science.

[7] Kapiteijn E, Putter H, van de Velde CJ (2002). Impact of the introduction and training of total mesorectal excision on recurrence and survival in rectal cancer in The Netherlands. Br J Surg.

[8] Riihimaki M, Hemminki A, Sundquist J, Hemminki K (2016). Patterns of metastasis in colon and rectal cancer. Sci Rep.

[9] Knijn N, van Erning FN, Overbeek LI, Punt CJ, Lemmens VE, Hugen N (2016). Limited effect of lymph node status on the metastatic pattern in colorectal cancer. Oncotarget.

[10] Nagtegaal ID, Knijn N, Hugen N, Marshall HC, Sugihara K, Tot T (2017). Tumor deposits in colorectal cancer: improving the value of modern staging—a systematic review and meta-analysis. J Clin Oncol.

[11] Lord AC, Knijn N, Brown G, Nagtegaal ID (2020). Pathways of spread in rectal cancer: a reappraisal of the true routes to distant metastatic disease. Eur J Cancer.

[12] Talbot IC, Ritchie S, Leighton MH, Hughes AO, Bussey HJ, Morson BC (1980). The clinical significance of invasion of veins by rectal cancer. Br J Surg.

[13] Wang Z, Zhou Z, Wang C, Zhao G, Chen Y, Gao H (2005). Microscopic spread of low rectal cancer in regions of the mesorectum: detailed pathological assessment with whole-mount sections. Int J Colorectal Dis.

[14] Hogan AM, Winter DC (2009). Complete mesocolic excision—a marker of surgical quality?. J Gastrointest Surg.

[15] Munkedal DLE, Rosenkilde M, Nielsen DT, Sommer T, West NP, Laurberg S (2017). Radiological and pathological evaluation of the level of arterial division after colon cancer surgery. Colorectal Dis.

[16] West NP, Kobayashi H, Takahashi K, Perrakis A, Weber K, Hohenberger W (2012). Understanding optimal colonic cancer surgery: comparison of Japanese D3 resection and European complete mesocolic excision with central vascular ligation. J Clin Oncol.

[17] Killeen S, Mannion M, Devaney A, Winter DC (2014). Complete mesocolic resection and extended lymphadenectomy for colon cancer: a systematic review. Colorectal Dis.

[18] Merkel S, Weber K, Matzel KE, Agaimy A, Gohl J, Hohenberger W (2016). Prognosis of patients with colonic carcinoma before, during and after implementation of complete mesocolic excision. Br J Surg.

[19] Bertelsen CA, Neuenschwander AU, Jansen JE, Kirkegaard-Klitbo A, Tenma JR, Wilhelmsen M (2016). Short-term outcomes after complete mesocolic excision compared with 'conventional' colonic cancer surgery. Br J Surg.

[20] Bertelsen CA, Kirkegaard-Klitbo A, Nielsen M, Leotta SM, Daisuke F, Gogenur I (2016). Pattern of colon cancer lymph node metastases in patients undergoing central mesocolic lymph node excision: a systematic review. Dis Colon Rectum.

